# Blood protein biomarkers for rheumatoid arthritis patients complicated by interstitial lung disease

**DOI:** 10.3389/fmed.2026.1890259

**Published:** 2026-08-12

**Authors:** Hiroshi Furukawa, Yuichi Hikichi, Kohei Kunieda, Shomi Oka, Takashi Higuchi, Kota Shimada, Atsushi Hashimoto, Akiko Komiya, Toshihiro Matsui, Naoshi Fukui, Shigeto Tohma, Kenji Itoh

**Affiliations:** 1 Department of Rheumatology, NHO Tokyo National Hospital, Kiyose, Japan; 2 Clinical Research Center for Allergy and Rheumatology, NHO Sagamihara National Hospital, Sagamihara, Japan; 3 Tuning Fork Bio Japan, MITSUI LINK-Lab SHINKIBA 2, Tokyo, Japan; 4 Department of Rheumatology, NHO Sagamihara National Hospital, Sagamihara, Japan; 5 Department of Rheumatic Diseases, Tokyo Metropolitan Tama Medical Center, Fuchu, Japan; 6 Department of Internal Medicine, Sagami Seikyou Hospital, Sagamihara, Japan; 7 Department of Clinical Laboratory, NHO Sagamihara National Hospital, Sagamihara, Japan; 8 Department of Life Sciences, Graduate School of Arts and Sciences, The University of Tokyo, Tokyo, Japan

**Keywords:** interstitial lung disease, lung, protein biomarker, proteomics, rheumatoid arthritis

## Abstract

**Objective:**

The prognosis of patients with rheumatoid arthritis (RA) complicated with interstitial lung disease (ILD) is very poor. We used blood protein profiles to identify biomarkers for ILD in patients with RA.

**Methods:**

Proteome analyses of plasma samples from 16 RA patients with ILD or without chronic lung disease (CLD) were performed with a proteomic assay using antibody immunoassay and next generation sequencing. Validation of the results was achieved by multiplex bead-based immunoassay of 20 candidate protein biomarkers in serum of 96 RA patients with ILD or without CLD.

**Results:**

Levels of carbohydrate antigen 125 (CA125), C-X-C motif chemokine ligand (CXCL)9, CXCL13, interleukin (IL)-19, tissue inhibitor of metalloproteinase (TIMP)-1, TIMP-2, and TIMP-4 were increased and those of C-C motif chemokine ligand (CCL)3 were decreased in RA with ILD compared with those without CLD. Multiple logistic regression analysis of CXCL9, CXCL13, and TIMP-1 was performed to generate an ILD-index. The area under the curve value of receiver operating characteristic curve of ILD-index was 0.818 (95% confidence interval 0.730–0.907).

**Conclusions:**

Blood biomarker profiles could represent diagnostic biomarkers for ILD in RA.

## Introduction

Interstitial lung disease (ILD), which frequently complicates rheumatoid arthritis (RA), an autoimmune disease that affects synovial joints with extra-articular manifestations (1), can influence the prognosis of RA patients (2, 3). Currently known biomarkers of ILD [surfactant protein-D (SP-D), Krebs von den lungen-6 (KL-6)] have high cutoff levels for RA-associated ILD (RA-ILD) with poor sensitivity (4). Because radiological exposure could not be ignored during the examination of chest high resolution computed tomography, serum diagnostic biomarkers would be useful to select RA patients to be examined for chest high resolution computed tomography. If these biomarkers could be used as prognosis, severity, development or progression, or treatment-response biomarkers in future, it will be clinically useful. Of the biomarkers studied in idiopathic pulmonary fibrosis, few have been validated for RA-ILD. Thus, RA-ILD biomarkers are urgently required.

Levels of blood proteins have been comprehensively analyzed by mass spectrometry to elucidate pathological disorders and aid biomarker development. Recently, proteomic assays using modified single strand DNA (5) or antibody immunoassays and next generation sequencing (6, 7) have been developed. Low concentrations of blood proteins were detected with high specificity using these proteome assays and systematic investigations of blood proteins have been conducted to identify new biomarkers. However, few proteomic analyses have been conducted for RA-ILD (5, 8, 9). This study examined blood protein profiles of ILD in RA patients to identify biomarkers specific for RA-ILD.

## Materials and methods

### Patients

RA patients with chest computed tomography imaging (*n* = 96) recruited at Sagamihara Hospital or Tokyo Hospital satisfied the Rheumatoid Arthritis Classification Criteria (10) or American College of Rheumatology criteria for RA (11). The RA patients were categorized by chest computed tomography to diagnose usual interstitial pneumonia (UIP), nonspecific interstitial pneumonia (NSIP), or no chronic lung disease (CLD), as previously described (12–14). ILD consisted of patients with UIP or NSIP. Samples of plasma and serum were obtained from patients with RA.

Research Ethics Committees of Tokyo National Hospital (190010) and Sagamihara Hospital reviewed and approved this study. Written informed consent was given by patients in this study, which was performed in accord with the tenets of the Helsinki Declaration.

### Proteome analyses of RA patient plasma samples

Sixteen RA patients (*n* = 4 UIP, *n* = 4 NSIP, *n* = 8 without CLD [CLD(–)]) provided plasma samples for proteome assay analysis using an antibody immunoassay and next generation sequencing (Olink Explore HT, Olink Proteomics, Uppsala, Sweden), as previously described (6, 7). Normalized protein expression (NPX) values were used to quantify the relative protein levels in the plasma samples on a log2 scale.

### Data preprocessing and normalization

Although NPX values underwent internal normalization through the Olink pipeline, inspection of raw NPX distributions revealed substantial sample-to-sample variability, with notable differences in distributional shapes and cumulative density curves (Supplementary Figure S1). To mitigate this residual technical variability and harmonize distributions across samples, quantile normalization was performed using Tuning-Base™, an in-house bioinformatics platform (15–17). The limits of detection were not set for Olink. No plate or batch effects were considered (Tuning Fork Bio Japan,Tokyo, Japan), because all the samples were analyzed in the same plate. Quantile normalization was applied across all discovery samples included in the proteome analysis, rather than separately within batches. No missing NPX values were present in the dataset used for candidate selection. No proteins were additionally excluded solely on the basis of low detection rate; all proteins passing the standard Olink output/QC were included in downstream analyses.

### Selection of candidate proteins associated with ILD subtypes

Because ILD exhibits marked inter individual heterogeneity, group level statistical comparisons may fail to capture biologically meaningful increases that occur only in a subset of patients. Therefore, instead of relying on significance testing, we used predefined threshold-based criteria designed to identify proteins showing a clear elevation in individual UIP or NSIP cases relative to CLD(–) (17).

A protein was considered a candidate when at least one sample in the UIP or NSIP group exceeded the disease group mean +2.5 standard deviation (SD), together with additional distribution-based thresholds as described below. A protein was considered UIP associated, if it met one or more of the following: Criterion 1 [≥1 UIP sample > (UIP mean +2.5 SD), UIP mean ≥ 5.169, UIP mean ≥ 2.5-fold CLD(–) mean, All CLD(–) samples < 5.169], Criterion 2 [≥1 UIP sample > (UIP mean +2.5 SD), UIP mean ≥ 5.169, UIP mean ≥ 4-fold CLD(–) mean, ≥ 80% of CLD(–) samples < 5.169], or Criterion 3 [≥1 UIP sample > (UIP mean +2.5 SD), UIP mean ≥ 7.771, UIP mean ≥ 4-fold CLD(–) mean, All CLD(–) samples < 7.771].

A protein was considered NSIP associated if it met one or more of the following: Criterion 1 [≥1 NSIP sample > (NSIP mean +2.5 SD), NSIP mean ≥ 7.771, NSIP mean ≥ 4-fold CLD(–) mean, All CLD(–) samples < 7.771], Criterion 2 [≥1 NSIP sample > (NSIP mean +2.5 SD), NSIP mean ≥ 5.169, NSIP mean ≥ 2-fold CLD(–) mean, All CLD(–) samples < 5.169], or Criterion 3 [≥1 NSIP sample > (NSIP mean +2.5 SD), NSIP mean ≥ 10.37, NSIP mean ≥ 2-fold CLD(–) mean, ≥ 67% of CLD(–) samples < 5.169]. The numerical thresholds were selected by a pre-specified exploratory algorithm to retain a tractable number of candidate proteins for downstream validation, while requiring clear elevation in UIP or NSIP samples and separation from CLD(–) controls. These thresholds were not intended as optimized diagnostic cutoffs, but as screening criteria for hypothesis generation.

### Unsupervised clustering using elevated proteins

To visualize subtype-associated patterns, we performed hierarchical clustering using the 459 proteins that were identified as elevated in UIP or NSIP relative to CLD(–) according to the predefined threshold-based criteria. Clustering was conducted on quantile-normalized NPX values, using Ward's minimum variance linkage and the Euclidean distance. Sample-wise dendrograms and the corresponding heat map are shown in Supplementary Figure S5.

### Measurement of proteins in RA patient serum samples

A multiplex bead-based immunoassay, Bio-Plex suspension array system (Bio-Rad, Hercules, CA, USA), was used to detect proteins in individual serum samples obtained from 96 RA patients who had CLD [CLD(+)] or were CLD(–). Human TIMP Luminex Performance Assay kits (R&D Systems, Minneapolis, MN, USA) were used to detect tissue inhibitor of metalloproteinase (TIMP)-1, TIMP-2, and TIMP-4. Luminex Human Discovery Assays (R&D Systems) were used to detect (Plex1) carbohydrate antigen 125 (CA125), C-X-C motif chemokine ligand (CXCL)9, CXCL10, CXCL11, CXCL13, interleukin (IL)-7, IL-17C, IL-33, C-C motif chemokine ligand (CCL)7, CCL26, IL-5, IL-11, IL-19, and matrix metalloproteinase (MMP)-12, (Plex2) CCL3, and tumor necrosis factor (TNF)α, and (Plex3) von Willebrand factor A2 domain (vWF-A2). Sera were diluted 1:2 for Plex1 and Plex2, 1:50 for Plex3, and 1:200 for TIMP. Values below the lower limit of quantification were calculated as zero.

### Statistical analysis

Differences related to the characteristics of RA patients were analyzed by t-test or Fisher's exact test with 2 × 2 contingency tables. Hierarchical cluster analysis was achieved using the Euclidean distance and Ward's method. Then, these were used to generate heatmaps with dendrograms. Principal component analysis was performed to discriminate between subgroups of RA patients. A comparison of protein amounts in sera from RA subsets and RA CLD(–) was performed using *t*-tests and correction for multiple testing was performed by calculating false discovery rate *Q*-value (18). Correlations between protein biomarker concentrations in proteome analyses and multiplex bead-based immunoassays were evaluated; Pearson's correlation coefficients were calculated and the null hypotheses were tested. Multiple logistic regression analyses of CXCL9, CXCL13, and TIMP-1 were used to generate an ILD-index. Multiple logistic regression analyses were also performed to assess independent association of ILD-index from clinical information and the individuals with missing clinical information were not used. Protein levels and the ILD-index were compared between RA with ILD [ILD(+)] and RA CLD(–) on receiver operating characteristic (ROC) curves. area under the curve (AUC) values for ROC curves with 95% confidence intervals (CIs) were determined, then a comparison with other ROC curves by chi-square tests. Estimations of optimized cut-off levels were determined based on the highest Youden index value. Monte Carlo cross-validation was conducted 100 rounds and the average AUC value for ROC curves was calculated to estimate the performance of the logistic regression model (19). Statistical significance was considered when *P* or *Q* < 0.05.

## Results

### Characteristics of RA patients

Table 1 presents the clinical manifestations of RA patients. The male to female ratio, percentage of ever smokers, mean age, and levels of KL-6, SP-D, and rheumatoid factor were higher in RA patients with UIP than in CLD(–). The mean age, male to female ratio, and percentage of ever smokers were higher in RA patients with NSIP than in CLD(–).

**Table 1:** Clinical manifestations of patients with RA.

Clinical manifestations	UIP (*n* = 25)	*P*	NSIP (*n* = 25)	*P*	CLD(-) (*n* = 46)
Age, years (SD)	75.0 (8.9)	3.47 × 10^−5^	68.6 (8.0)	0.0381	63.0 (11.9)
Male *n* (%)	10.0 (40.0)	0.0008	9.0 (36.0)	0.0022	2.0 (5.1)
Disease duration, years (SD)	9.0 (12.7)	0.4402	9.6 (8.3)	0.5251	11.3 (11.0)
Steinbrocker stage 3, 4, *n* (%)	10.0 (43.5)	0.7951	5.0 (21.7)	0.1840	17.0 (38.6)
Smoker or past smoker, *n* (%)	12.0 (54.5)	0.0014	10.0 (50.0)	0.0031	3.0 (10.3)
KL-6, U/mL (SD)	1097.6 (1070.0)	0.0015	619.5 (834.3)	0.0913	298.4 (142.9)
SP-D, ng/mL (SD)	174.7 (121.1)	0.0059	125.2 (192.9)	0.3473	75.9 (65.0)
RF, U/mL (SD)	276.6 (441.7)	0.0175	200.4 (264.5)	0.0502	103.9 (144.3)
ACPA, U/mL (SD)	216.7 (208.9)	0.4922	294.4 (275.2)	0.9308	306.1 (626.7)
Corticosteroid administration, *n* (%)	16 (69.6)	1.74 × 10^−6^	12 (48.0)	0.0009	4 (10.0)
csDMARDs administration, *n* (%)	14 (93.3)	0.3295	21 (100.0)	0.0556	11 (78.6)
b/tsDMARDs administration, *n* (%)	5 (50.0)	0.0043	1 (6.3)	1.0000	2 (6.1)

RA, rheumatoid arthritis; UIP, usual interstitial pneumonia; NSIP, nonspecific interstitial pneumonia; CLD, chronic lung disease; KL-6, Krebs von den Lungen-6; SP-D, Surfactant Protein-D; RF, Rheumatoid factor; ACPA, anti-citrullinated peptide antibody; csDMARDs, conventional synthetic disease-modifying antirheumatic drugs; b/tsDMARDs, biological or targeted synthetic disease-modifying antirheumatic drugs. Data are presented as the mean value or number of each group. Phenotype frequencies or standard deviations were shown in parenthesis. Differences were evaluated for comparisons with the CLD(–) population by *t*-test or Fisher's exact test using 2 × 2 contingency tables.

### Proteome analyses

**Figure 1:**
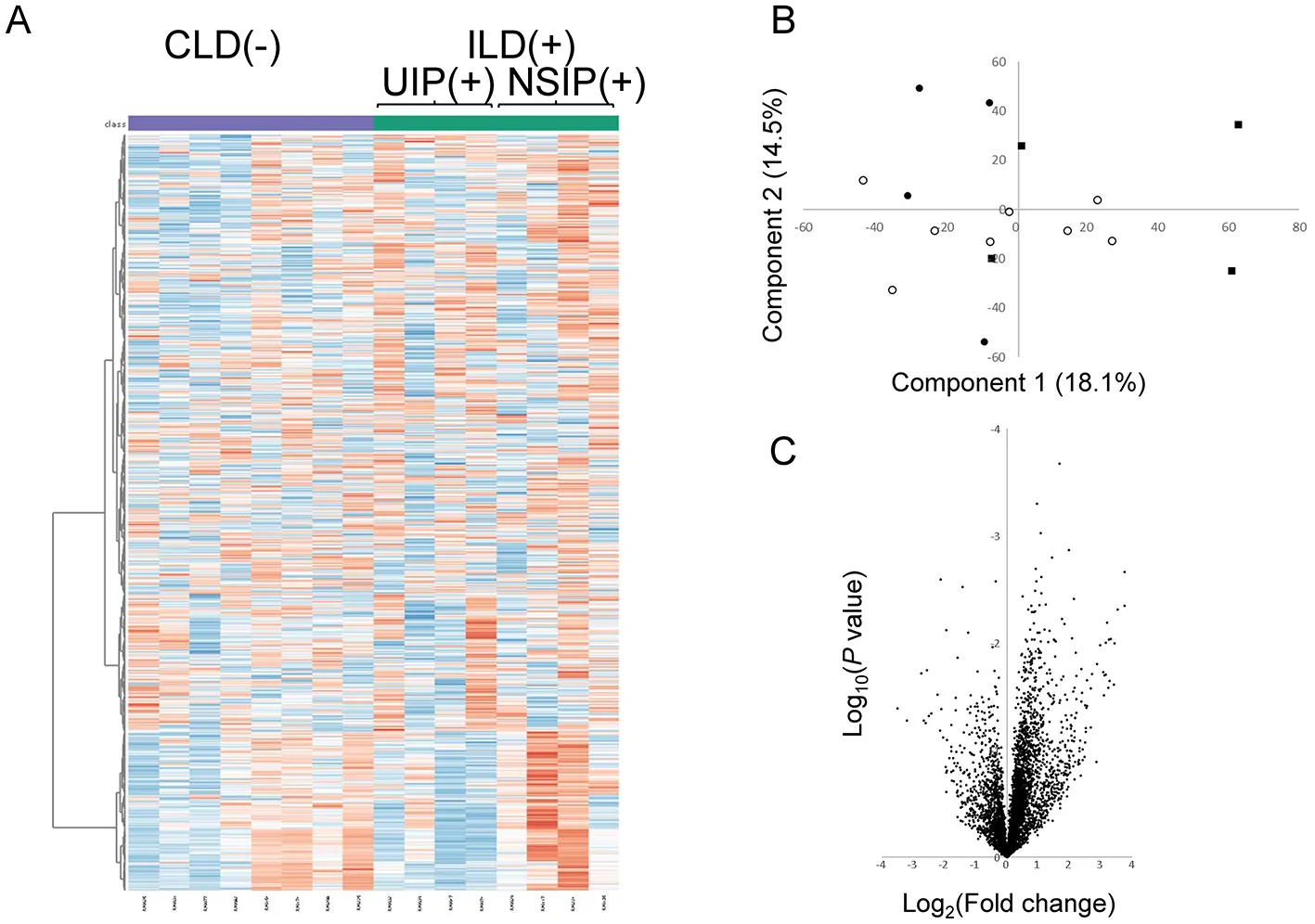
Results of hierarchical clustering, principal component, and volcano plot analyses on protein biomarkers of 16 plasma samples from RA participants [*n* = 4 UIP, *n* = 4 NSIP, and *n* = 8 CLD(–)]. **(A)** Hierarchical clustering analysis of protein biomarkers. Each column and row indicate a sample from an RA participant and a protein biomarker, respectively. **(B)** Scores plots for principal component analyses of protein biomarkers. Filled circles, filled squares, and empty circles represent RA with UIP, RA with NSIP, and RA without CLD, respectively. **(C)** The volcano plot shows the differences in protein biomarkers between RA participants with ILD or without CLD. RA, rheumatoid arthritis; ILD, interstitial lung disease; UIP, usual interstitial pneumonia; NSIP, nonspecific interstitial pneumonia; CLD, chronic lung disease.

Proteome analyses of 16 plasma samples from RA patients [*n* = 4 UIP, *n* = 4 NSIP, and *n* = 8 CLD(–)] were performed. The raw NPX values are shown in Supplementary Table S1. Hierarchical clustering with visualization on a heat map for all proteins measured in Olink analyses was performed (Figure 1A). Principal component analysis was conducted with the protein biomarkers to discriminate between subgroups of RA patients, but this suggested the absence of subgroups of RA (Figure 1B). NPX values of protein biomarkers in plasma from RA ILD(+) were compared with those from RA CLD(–) (Figure 1C). The smallest *P* value observed was 2.12 × 10^−4^ for SRGAP3, which was higher than 9.23 × 10^−6^: 0.05 divided by the measured protein number 5,420. Thus, no protein significantly associated with ILD in RA was detected in the proteome analyses.

### Identification of candidate proteins associated with ILD subtypes

To characterize protein markers associated with ILD development in RA, we compared the serum profiles of UIP and NSIP against CLD(–) using predefined threshold-based criteria as described in the Materials and methods. Because ILD exhibits substantial inter individual heterogeneity, we prioritized identifying proteins with clear elevations in individual patients rather than testing for group level statistical differences.

Candidate proteins were identified in UIP relative to CLD(–): 98 proteins met Criterion 1, 131 proteins met Criterion 2, and 178 proteins met Criterion 3. The overlaps among these protein sets are summarized in a Venn diagram (Supplementary Figure S3A), and full lists of proteins identified under each criterion are provided in Supplementary Tables S2–S5.

Candidate proteins were also identified in NSIP relative to CLD(–): 116 proteins satisfied Criterion 1, 105 proteins satisfied Criterion 2, and 35 proteins satisfied Criterion 3. The relationships between the three NSIP associated protein sets are presented in Supplementary Figure S3B, and detailed protein lists are available in Supplementary Tables S6–S9.

### Overall pattern of ILD-associated proteins

Across both UIP and NSIP analyses, a substantial number of proteins showed a marked elevation relative to CLD(–). Although each criterion captured partially overlapping but distinct subsets of proteins, together they highlight the heterogeneous molecular signatures characteristic of UIP and NSIP (Supplementary Figure S4). When hierarchical clustering was restricted to the 459 proteins elevated in UIP or NSIP, the samples clearly separated CLD(–) from ILD (UIP/NSIP), in contrast to the weaker separation observed when using all measured proteins (Supplementary Figure S4). This supports the notion that distribution-based selection enriches subtype-informative signals despite substantial inter-individual heterogeneity. This threshold-based approach was used to identify proteins that were prominently elevated in specific patients, reflecting the individualized nature of ILD biology.

Candidate protein markers were selected from the results of these proteome analyses. The 18 proteins analyzed by multiplex bead-based immunoassays were selected from the candidate proteins in Supplementary Tables S2–S9 and additional markers including TIMP-1 and TIMP-4 were also analyzed, because they were included in the kit to detect TIMP-2. Thus, 20 candidate protein markers were selected to confirm the results of proteome analyses.

### Serum protein biomarker profiles of RA ILD(+)

**Figure 2:**
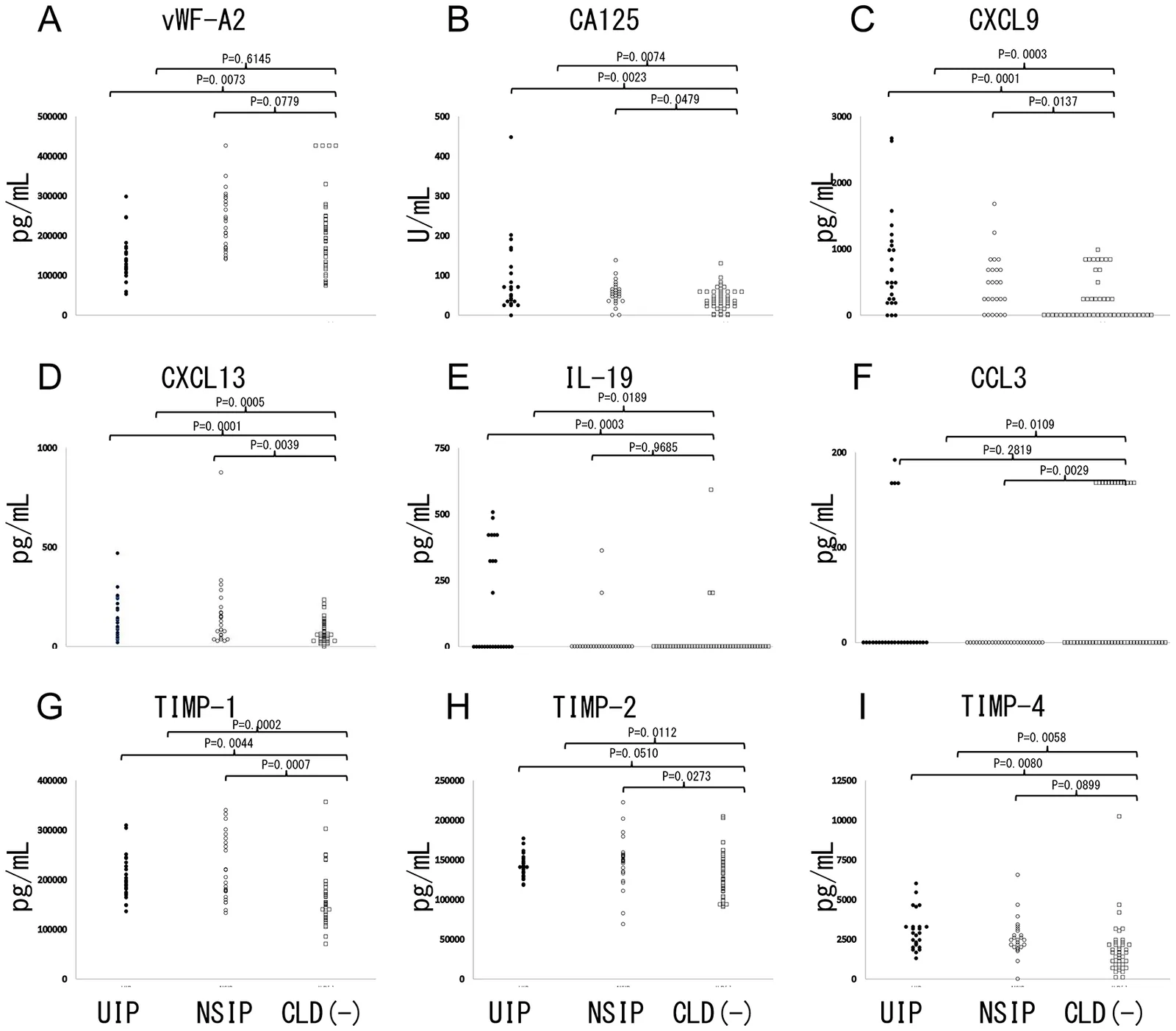
Validation of protein biomarker profiles of RA patients. Distribution of protein biomarkers are shown: **(A)** vWF-A2; **(B)** CA125; **(C)** CXCL9; **(D)** CXCL13; **(E)** IL-19; **(F)** CCL3; **(G)** TIMP-1; **(H)** TIMP-2; **(I)** TIMP-4. Filled circles, empty circles, and empty squares represent RA with UIP, RA with NSIP, and RA CLD(–), respectively. RA, rheumatoid arthritis; UIP, usual interstitial pneumonia; NSIP, nonspecific interstitial pneumonia; CLD, chronic lung disease; vWF-A2, von Willebrand factor A2 domain; CA125, carbohydrate antigen 125; CXCL, C-X-C motif chemokine ligand; IL, interleukin; CCL, C-C motif chemokine ligand; TIMP, tissue inhibitor of metalloproteinase.

These 20 candidate protein markers were validated with multiplex bead-based immunoassays to detect proteins in individual sera from 96 patients that included the patients used for the proteome analyses. The standard curves for multiplex bead-based immunoassay of 20 candidate protein biomarkers were shown in Supplementary Figure S5 and the validation results are shown in Table 2 and Supplementary Figure S6. The correlations between the results from proteome analyses of plasma samples and multiplex bead-based immunoassays of serum samples from 16 patients with RA were shown in Table 3 (average *r* = 0.35). The levels of CA125, CXCL9, CXCL13, IL-19, TIMP-1, TIMP-2, and TIMP-4 were increased and those of CCL3 were decreased in RA ILD(+) compared with CLD(–) (Figure 2). The levels of CA125, CXCL9, CXCL13, IL-19, MMP-12, TIMP-1, and TIMP-4 were increased and those of vWF-A2 were decreased in RA with UIP compared with CLD(–). The levels of CXCL13 and TIMP-1 were increased and those of CCL3 were decreased in RA with NSIP compared with CLD(–). Thus, the levels of some serum protein biomarkers were altered in RA ILD(+).

**Table 2:** Protein concentrations in serum samples from patients with RA.

Protein	ILD (*n* = 50)	*Q*	UIP (*n* = 25)	*Q*	NSIP (*n* = 25)	*Q*	CLD(-) (*n* = 46)
vWF-A2 (pg/mL)	190,614.43 (80,686.21)	0.7374	142,896.77 (56,167.19)	0.0218	238,332.08 (73,439.37)	0.2004	199,605.85 (93,567.63)
CA125 (U/mL)	71.46 (70.83)	0.0265	87.73 (93.64)	0.0104	55.19 (30.39)	0.1438	40.94 (27.39)
CXCL9/MIG (pg/mL)	613.50 (604.40)	0.0025	763.05 (721.60)	0.0013	463.95 (422.51)	0.0615	232.11 (336.52)
CXCL11/I-TAC (pg/mL)	26.39 (32.71)	0.8087	22.32 (33.79)	0.9265	30.47 (31.75)	0.6568	23.50 (58.63)
IL-7 (pg/mL)	16.51 (6.51)	0.4540	15.45 (6.69)	0.9511	17.57 (6.28)	0.2752	15.09 (7.58)
IL-17C (pg/mL)	0.00 (0.00)	NA	0.00 (0.00)	NA	0.00 (0.00)	NA	0.00 (0.00)
IL-33 (pg/mL)	35.68 (85.37)	0.4791	41.32 (100.77)	0.4261	30.05 (68.26)	0.6549	20.12 (64.46)
CCL7 (pg/mL)	0.00 (0.00)	NA	0.00 (0.00)	NA	0.00 (0.00)	NA	0.00 (0.00)
CCL26 (pg/mL)	10.51 (27.20)	0.4632	11.91 (36.48)	0.6810	9.10 (13.24)	0.6257	22.44 (87.31)
CXCL10 (pg/mL)	59.82 (55.93)	0.7973	48.42 (28.59)	0.6875	71.22 (72.80)	0.4601	55.62 (53.61)
CXCL13 (pg/mL)	155.20 (143.35)	0.0029	153.46 (107.08)	0.0008	156.94 (174.59)	0.0232	73.94 (53.64)
IL-5 (pg/mL)	0.00 (0.00)	0.4903	0.00 (0.00)	0.6438	0.00 (0.00)	0.5978	0.24 (1.60)
IL-11 (pg/mL)	30.85 (62.23)	0.2406	31.16 (76.34)	0.4154	30.53 (45.59)	0.2303	14.62 (39.24)
IL-19 (pg/mL)	88.41 (165.56)	0.0425	154.21 (200.73)	0.0020	22.62 (81.56)	0.9685	21.72 (95.68)
MMP-12 (pg/mL)	40.39 (42.30)	0.0745	48.24 (58.13)	0.0423	32.55 (12.07)	0.1806	26.43 (15.42)
CCL3 (pg/mL)	13.92 (47.77)	0.0326	27.84 (65.24)	0.4613	0.00 (0.00)	0.0257	47.43 (76.40)
TNF-alpha (pg/mL)	7.04 (7.46)	0.7760	6.30 (7.01)	0.9662	7.78 (7.96)	0.6187	6.54 (9.62)
TIMP-1 (pg/mL)	211,915.30 (55,100.91)	0.0027	203,947.52 (43,506.46)	0.0160	219,883.08 (64,603.80)	0.0119	163,894.79 (57,794.82)
TIMP-2 (pg/mL)	144,052.71 (25,068.54)	0.0289	141,466.76 (15,015.13)	0.1019	146,638.66 (32,305.77)	0.0984	129,479.92 (27,475.33)
TIMP-4 (pg/mL)	2,829.68 (1,220.18)	0.0261	3,032.18 (1,213.34)	0.0205	2,627.18 (1,217.41)	0.2023	1,942.08 (1,722.68)

RA, rheumatoid arthritis; ILD, interstitial lung disease; UIP, usual interstitial pneumonia, NSIP, nonspecific interstitial pneumonia; CLD, chronic lung disease; CLD(–), without CLD; CXCL. C-X-C motif chemokine ligand; TIMP, tissue inhibitor of metalloproteinase; CA125, carbohydrate antigen 125; CCL, C-C motif chemokine ligand; IL, interleukin; MMP, matrix metalloproteinase; vWF-A2, von Willebrand factor A2 domain; TNF, tumor necrosis factor; ILD, group includes UIP and NSIP groups; Average value of each group was shown. Standard deviations are shown in parenthesis. Differences were evaluated for comparisons with the CLD(–) population by *t*-test. Correction for multiple testing was performed by calculating false discovery rate *Q*-value. TIMP-1, TIMP-2, and TIMP-4 were analyzed in 38 CLD(–) RA patients and other proteins in 46 CLD(–) RA.

**Table 3:** Correlations between proteome analyses and multiplex bead-based immunoassays.

Protein	RA (*n* = 16)	
	*r*	*P*
vWF-A2	0.31	0.2429
CA125/MUC16	0.79	0.0003
CXCL9/MIG	0.64	0.0079
CXCL11/I-TAC	0.11	0.6905
IL-7	0.17	0.5348
IL-17C	NA	NA
IL-33	−0.07	0.7841
CCL7/MCP-3/MARC	NA	NA
CCL26/Eotaxin-3	0.88	8.41 × 10^−6^
CXCL10/IP-10/CRG-2	0.64	0.0072
CXCL13/BLC/BCA-1	0.89	3.79 × 10^−6^
IL-5	−0.06	0.8333
IL-11	−0.02	0.9332
IL-19	−0.19	0.4921
MMP-12	0.01	0.9817
CCL3/MIP-1 alpha	0.02	0.9318
TNF-alpha	0.41	0.1187
TIMP-1	0.44	0.0875
TIMP-2	0.60	0.0142
TIMP-4	0.71	0.0020
Average	0.35	

RA, rheumatoid arthritis; Correlations between the results from proteome analyses of plasma samples and multiplex bead-based immunoassays of serum samples from 16 patients with RA were evaluated. Pearson's correlation coefficients were calculated and the null hypotheses were tested.

### ROC curve analyses

**Figure 3:**
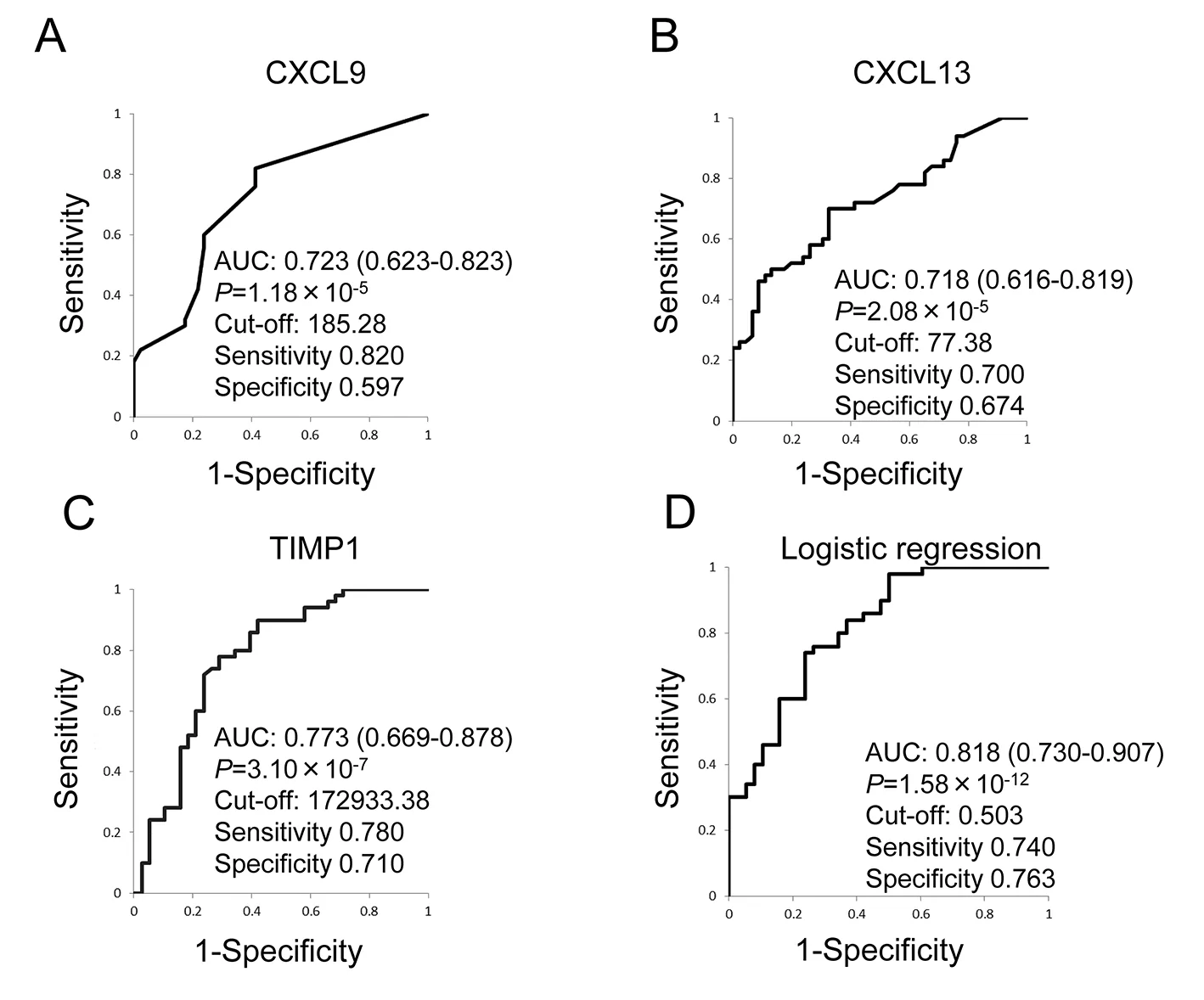
ROC curves using CXCL9, CXCL13, and TIMP-1 for comparisons between RA ILD(+) and RA CLD(–). ROC curves for CXCL9 **(A)**, CXCL13 **(B)**, TIMP-1 **(C)**, and ILD-index **(D)** are shown. The ILD-index was generated by multiple logistic regression analyses of CXCL9, CXCL13, and TIMP-1. The AUC values of the ROC curves with 95% confidence intervals and the optimized cut-off levels with specificities and sensitivities are described. RA, rheumatoid arthritis; ILD, interstitial lung disease; CLD, chronic lung disease; CLD(–),without CLD. ILD group includes usual interstitial pneumonia and nonspecific interstitial pneumonia groups. ROC, receiver operating characteristic; AUC, area under the curve; CXCL, C-X-C motif chemokine ligand; TIMP, tissue inhibitor of metalloproteinase.

The ROC curves for serum protein biomarkers with *Q-*values < 0.01 (CXCL9, CXCL13, and TIMP-1) were generated for comparisons between RA ILD(+) and RA CLD(–) (Figures 3A–C). Multiple logistic regression analyses of these three serum protein biomarkers were performed to generate an ILD-index = 0.0015 × (CXCL9) + 0.0085 × (CXCL13) + 1.37 × 10^−5^ × (TIMP-1)-−3.6664. The AUC value of the ROC curve of ILD-index was 0.818 (95% CI 0.730–0.907, Figure 3D), which was higher than that for CXCL13 (*P* = 0.0340), but not for CXCL9 (*P* = 0.0649), TIMP-1 (*P* = 0.2745), KL-6 (*P* = 0.2793, Supplementary Figure S7A), or SP-D (*P* = 0.1079, Supplementary Figure S7B). The average AUC value for ROC curves generated by cross-validation was 0.789 [95% confidence interval (CI) 0.658–0.908]. To eliminate effects of the different clinical characteristics of RA patients with ILD and without CLD, multiple logistic regression analyses were also performed (Table 4). An association of ILD with ILD-index remained (*P*_adjusted_ = 0.0316, OR_adjusted_ 28.56, 95% CI 1.34–607.39), when conditioned on the clinical characteristics, suggesting ILD-index was independently associated with ILD.

**Table 4:** Multiple logistic regression analysis of ILD-index and clinical manifestations for ILD in RA.

Variable	Unconditioned			Conditioned on the other factors		
	OR	95% CI	*P*	OR_adjusted_	95% CI	*P*_adjusted_
ILD-index	142.10	(17.26–1,169.95)	4.07 × 10^−6^	28.56	(1.34–607.39)	0.0316
Age, years	1.09	(1.04–1.14)	0.0004	1.06	(0.97–1.14)	0.1859
Male	2.52	(1.00–6.36)	0.0504	4.17	(0.69–25.01)	0.1185
Disease duration, years	0.98	(0.94–1.02)	0.3809	0.96	(0.89–1.04)	0.3007
Steinbrocker stage	0.85	(0.61–1.18)	0.3372	1.01	(0.48–2.12)	0.9796
Smoker or past smoker	2.04	(0.84–4.97)	0.1149	3.54	(0.64–19.49)	0.1458
RF, IU/ml	1.00	(1.00–1.01)	0.0333	1.00	(1.00–1.00)	0.6265
ACPA, U/ml	1.00	(1.00–1.00)	0.5991	1.00	(1.00–1.00)	0.3348
Corticosteroid administration, *n* (%)	5.04	(2.04–12.47)	0.0005	13.74	(2.29–82.61)	0.0042
csDMARDs administration, *n* (%)	0.57	(0.21–1.53)	0.2626	1.68	(0.18–15.47)	0.6490
b/tsDMARDs administration, *n* (%)	2.40	(1.01–5.72)	0.0486	4.99	(0.71–34.93)	0.1054

RA, rheumatoid arthritis; ILD, interstitial lung disease; RF, Rheumatoid factor; ACPA, anti-citrullinated peptide antibody; csDMARDs, conventional synthetic disease-modifying antirheumatic drugs; b/tsDMARDs, biological or targeted synthetic disease-modifying antirheumatic drugs; OR, odds ratio; CI, confidence interval. *P*, OR, 95% CI, *P*_adjusted_, and OR_adjusted_ were calculated by logistic regression analysis of RA patients.

## Discussion

Biomarker profiles as well as the roles of MMPs, chemokines, and serine protease inhibitors in idiopathic pulmonary fibrosis have been reported previously (20). However, few proteomic studies have investigated the roles of MMPs, vWF, cytokines, or chemokines in RA-ILD (5, 8, 9). These studies reported MMP-3, MMP-7, vWF, CCL21, KL-6, and IL-7 were candidate protein markers. Several candidate protein markers, including MMP-7, CCL18, KL-6, SP-D, CA125, and CXCL10, have been evaluated for RA-ILD using a candidate protein approach (21–25). In the present study, CXCL9, CXCL13, and TIMP-1 were found to be diagnostic biomarkers for RA-ILD, and the complex biomarker ILD-index was better at distinguishing ILD in RA.

The cutoff levels of these existing biomarkers, KL-6 and SP-D, were set for idiopathic pulmonary fibrosis and ILD in collagen diseases (23, 26) and were higher for their application to RA-ILD. The sensitivities are not sufficient (4). Additionally, the AUC value of the ROC curve of ILD-index was comparable to those of KL-6 or SP-D.

CXCL9 was previously predicted to be a plasma protein biomarker for idiopathic pulmonary fibrosis (27). A previous study reported serum levels of TIMP-1 were significantly increased in acute-onset diffuse ILD (AoDILD), which occurs in RA patients (28). In our study, serum levels of TIMP-1 were significantly increased in RA with ILD using a Luminex assay. In the Olink analyses, the levels of TIMP-1 also tended to increase in RA with ILD, although it was not selected using our selection methods. TIMP-1 is a member of the MMP inhibitor family, including TIMP-1, -2, -3, and -4. TIMP-1 is produced in most tissues, especially in artery and lung (https://gtexportal.org/home/gene/TIMP1), and inhibits the functions of MMP including the degradation of the structural proteins of extracellular matrix components and tissue remodeling. The roles of TIMP-1 expressed in lung fibroblasts were suggested for the progression in ILD (29). However, the increased levels of CXCL9 or CXCL13 in RA ILD(+) were not reported in previous studies, but the increased levels of these chemokines were found in ILD associated with collagen diseases (30–32). Although many immune- and inflammation-related proteins were enriched in the Olink platforms, elevations of the immune- and inflammation-related proteins, CXCL9 and CXCL13, were confirmed in RA ILD(+) by Luminex assay. CXCL9 and CXCL13 are expressed in epithelial cells and macrophages. CXCL9 interacts with CXCR3 to regulate the migration, activation, and differentiation of macrophages, cytotoxic lymphocytes, or natural killer cells (33). CXCL13 interacts with CXCR5 to regulate the organization of B cells in lymph follicles (34). This information suggested that several types of immunological cells are involved in the pathogenesis of ILD in RA. The functional interactions of these protein biomarkers and these producing cells could be revealed using multi-omics data analyses. Targeting cytokine pathways—either supplementing those that are decreased or blocking those that are increased—may represent potential therapeutic strategies for RA-ILD. Serum levels of CXCL9 were reported to be correlated with those of bronchoalveolar lavage fluid (30). Although the expression of these protein biomarkers in lung tissues or bronchoalveolar lavage fluid was not detected in the present study, it should be confirmed in future studies. The expression levels of CA125, IL-19 and MMP-12 were increased in RA with UIP, but not in RA with NSIP, suggesting the roles of CA125, IL-19 and MMP-12 in the pathogenesis of UIP in RA.

If these biomarkers could be used for the prediction of prognosis, severity, development or progression, or treatment-response of ILD in RA, it will be clinically useful. Serum levels of CXCL9 before the treatment were correlated with the treatment-response of ILD associated with collagen diseases (30). Thus, future longitudinal studies on the prediction of prognosis, severity, development or progression, or drug efficacy of ILD in RA should be conducted.

The results of proteome analyses were not strongly correlated with multiplex bead-based immunoassays. Plasma samples were used for proteome analyses and serum samples were used for multiplex bead-based immunoassays. Expression levels of some proteins are different between serum and plasma samples. However, the difference of platforms might predominantly influence the results (35), suggesting that biomarker proteins confirmed in different detection methods and sample types would be robust.

In this study, group level statistical comparisons failed to capture statistical significance from proteomic analyses results and threshold-based criteria were used for the selection of candidate protein markers. In the validation analyses, CXCL9, CXCL13, and TIMP-1 were found to be diagnostic biomarkers for RA-ILD. These results indicate RA-ILD and idiopathic pulmonary fibrosis might have different pathogenic pathways. A study limitation was the small sample size. Because the sample size on the proteome analyses was small, validation with multiplex bead-based immunoassays was performed in larger sample size. However, the sample size of the validation was still modest. Therefore, the profiles of these biomarkers should be confirmed in future large-scale studies.

## Data Availability

The original contributions presented in this study are included in the article/Supplementary material, further inquiries can be directed to the corresponding author.
