# Supplementary material for: Blood protein biomarkers for rheumatoid arthritis patients complicated by interstitial lung disease
Source: Front Med (Lausanne). 2026 Aug 12;13:1890259. doi: 10.3389/fmed.2026.1890259 (PMC13509132; doi:10.3389/fmed.2026.1890259)
Supplement: Supplementary file 1 [file Data_Sheet_1.pdf]

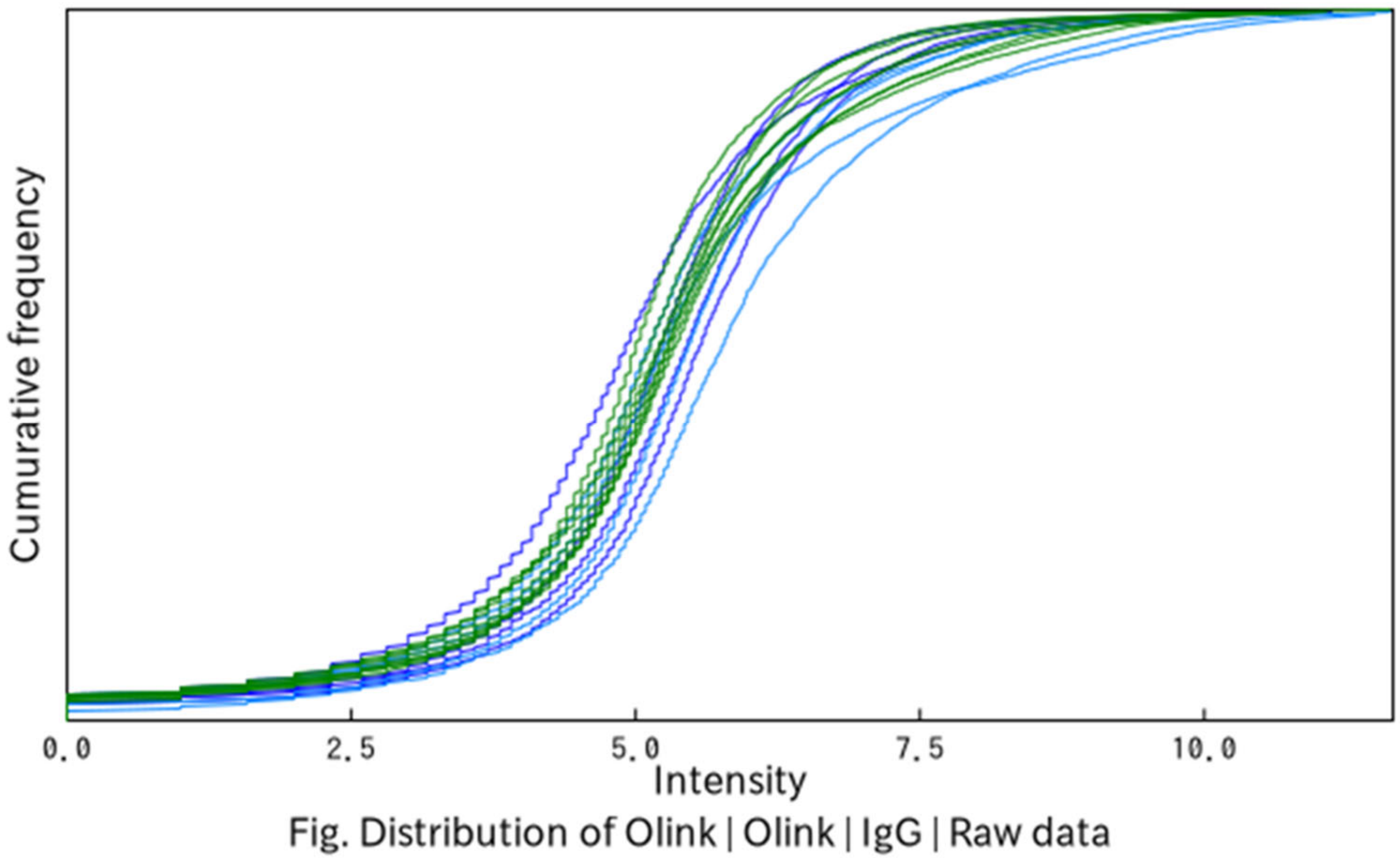

Supplementary Figure S1. Cumulative distribution plots of raw NPX values from all samples prior to normalization. The curves show that the NPX distributions differed markedly between samples, indicating residual technical variability after standard Olink processing. These plots were generated using the Tuning-Base™ software and motivated the application of quantile normalization to harmonize sample wise distributions before downstream analysis. NPX: normalized protein expression.
