# Supplementary material for: Blood protein biomarkers for rheumatoid arthritis patients complicated by interstitial lung disease
Source: Front Med (Lausanne). 2026 Aug 12;13:1890259. doi: 10.3389/fmed.2026.1890259 (PMC13509132; doi:10.3389/fmed.2026.1890259)
Supplement: Supplementary file 2 [file Data_Sheet_2.pdf]

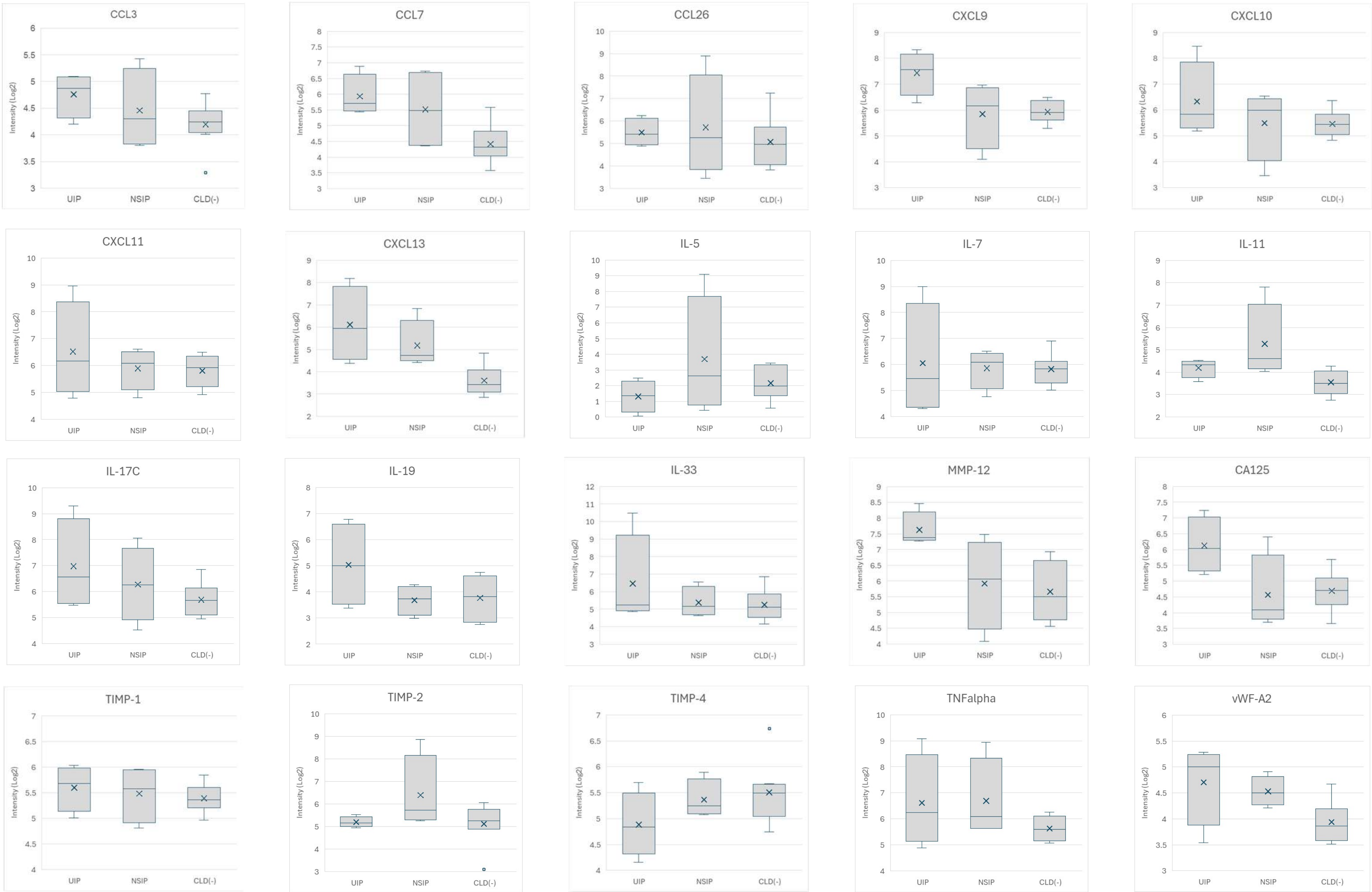

Supplementary Figure S2. Box-and-whisker plots of protein biomarker intensities in RA subgroups. Box-and-whisker plots showing quantile-normalized NPX (log2) intensities for each protein across UIP (n = 4), NSIP (n = 4), and CLD(−) (n = 8). The center line indicates the median; boxes represent the interquartile range (IQR); whiskers denote the minimum and maximum values excluding outliers. Outliers are plotted as individual points and defined as values outside the range Q1 − 1.5 × IQR to Q3 + 1.5 × IQR. Intensities are displayed on a log2 scale. NPX values were quantile-normalized using Tuning-Base™ prior to visualization. RA, rheumatoid arthritis; ILD, interstitial lung disease; CLD(−), without chronic lung disease; UIP, usual interstitial pneumonia; NSIP, nonspecific interstitial pneumonia; NPX, normalized protein expression; IQR, interquartile range.
