# Supplementary material for: Blood protein biomarkers for rheumatoid arthritis patients complicated by interstitial lung disease
Source: Front Med (Lausanne). 2026 Aug 12;13:1890259. doi: 10.3389/fmed.2026.1890259 (PMC13509132; doi:10.3389/fmed.2026.1890259)
Supplement: Supplementary file 3 [file Data_Sheet_3.pdf]

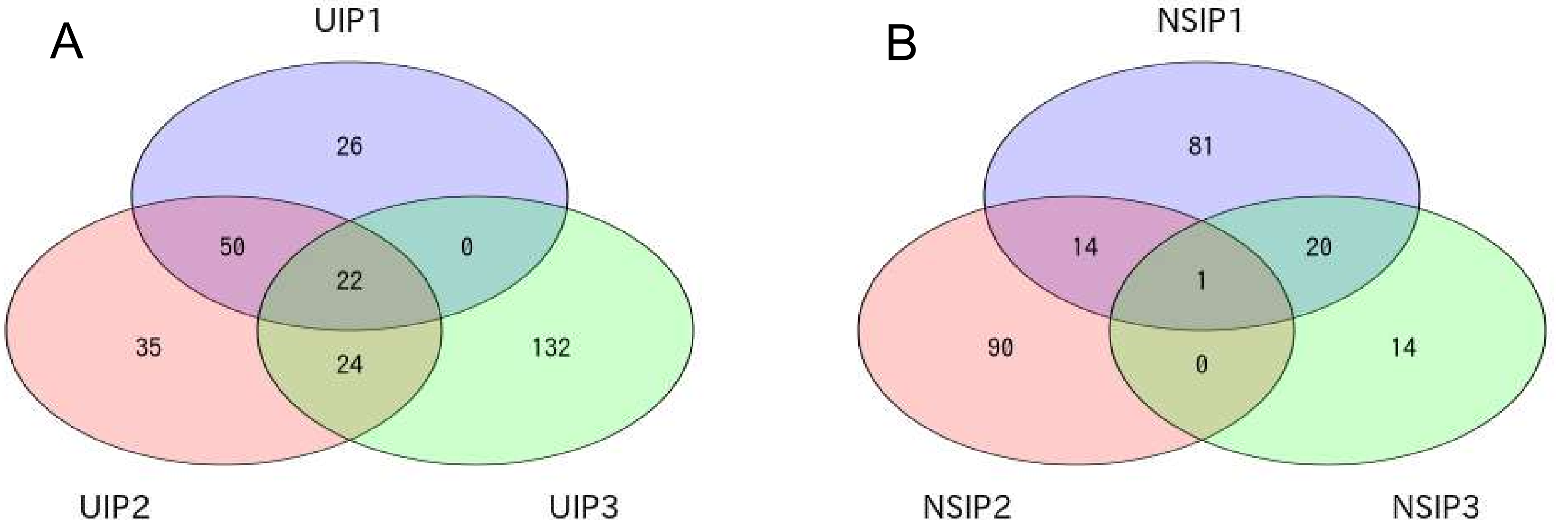

Supplementary Figure S3. Protein biomarker candidates of RA patients with UIP or NSIP. Protein biomarker candidate numbers in RA with UIP (A) or NSIP (B). RA: rheumatoid arthritis, UIP: usual interstitial pneumonia, NSIP: nonspecific interstitial pneumonia, CLD: chronic lung disease.
