# Supplementary material for: Blood protein biomarkers for rheumatoid arthritis patients complicated by interstitial lung disease
Source: Front Med (Lausanne). 2026 Aug 12;13:1890259. doi: 10.3389/fmed.2026.1890259 (PMC13509132; doi:10.3389/fmed.2026.1890259)
Supplement: Supplementary file 4 [file Data_Sheet_4.pdf]

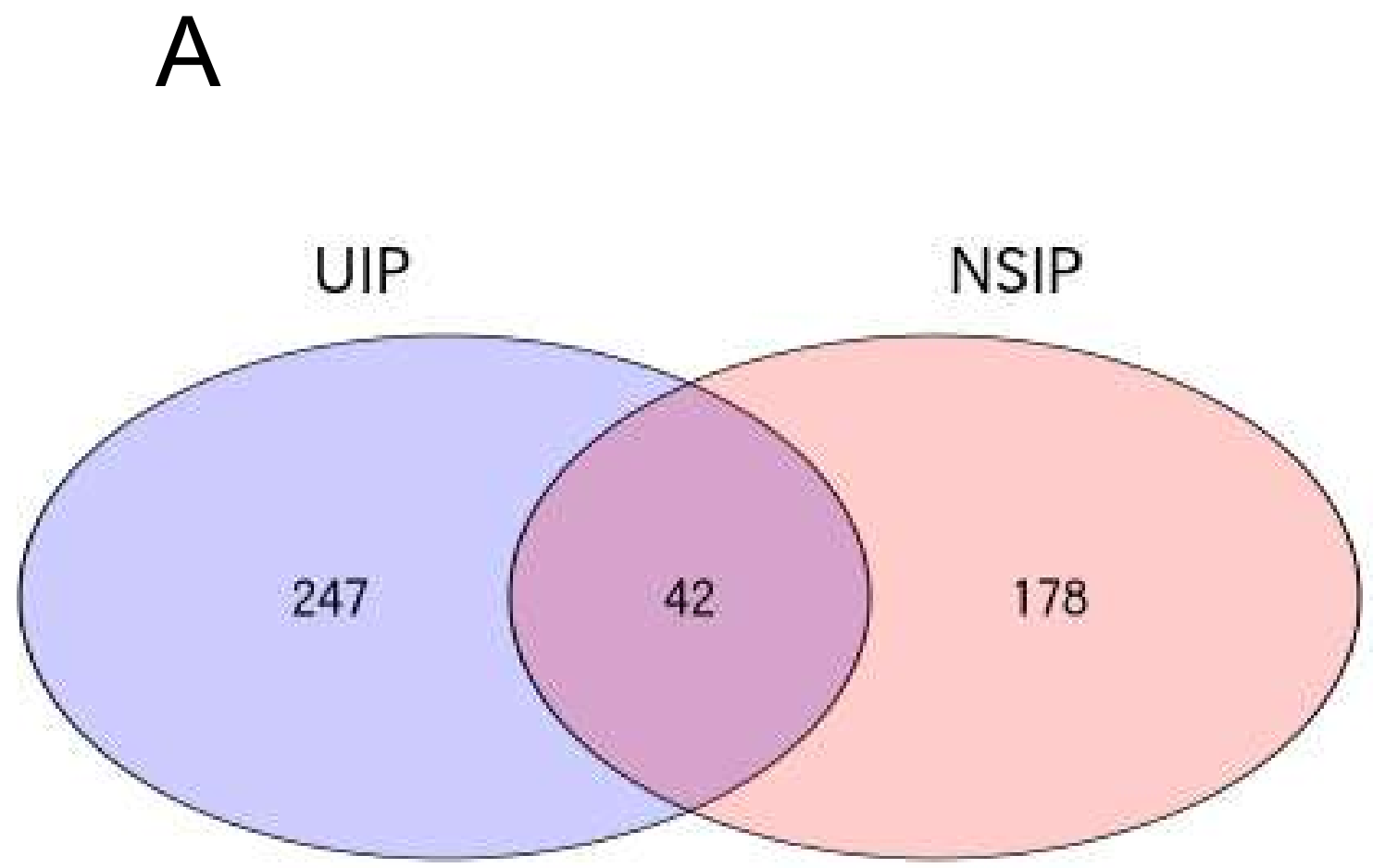

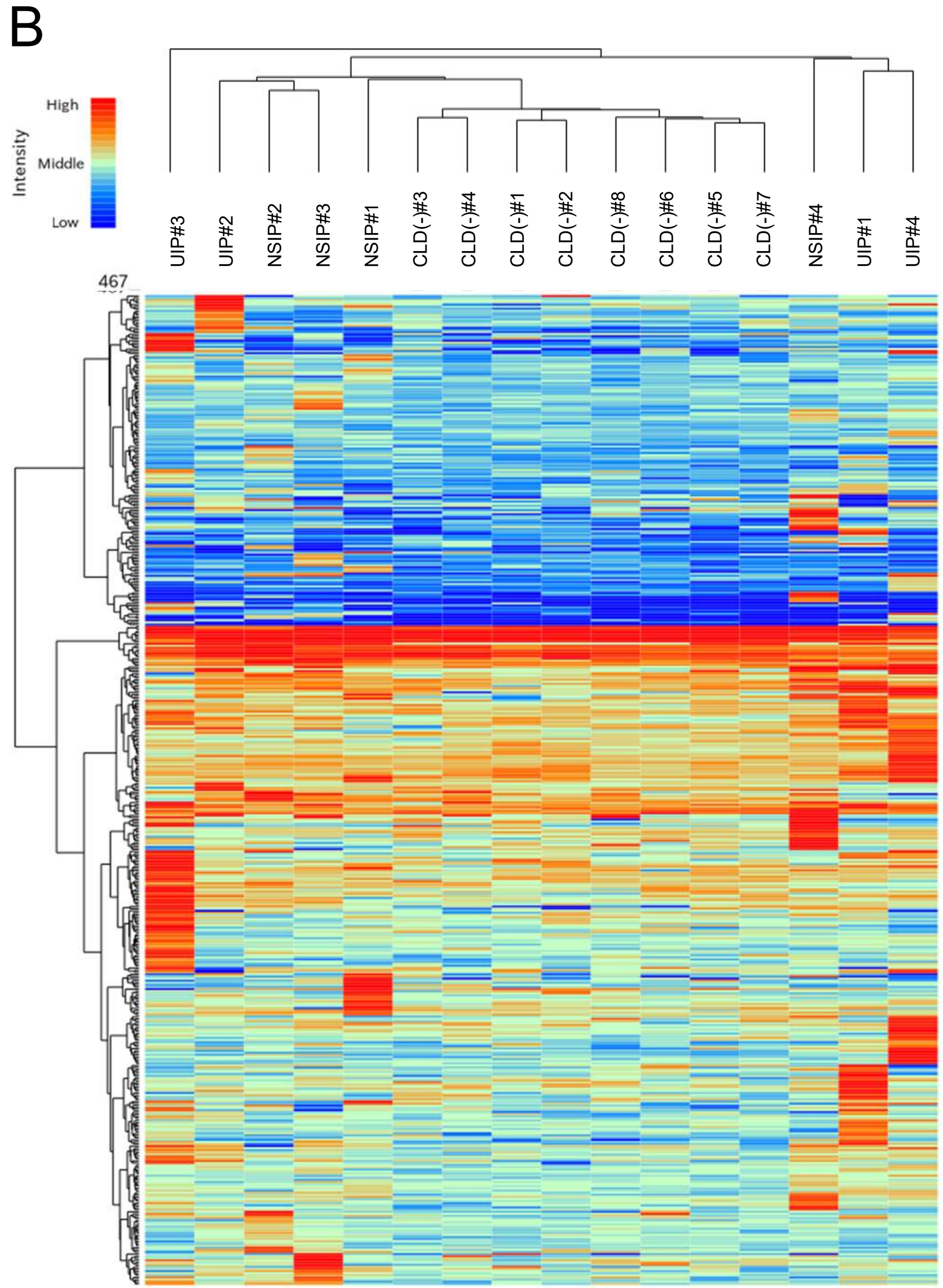

Supplementary Figure S4. Hierarchical clustering using proteins elevated in UIP or NSIP. Protein biomarker candidate numbers in RA with UIP or NSIP (A). Heat map and dendrograms based on 459 proteins elevated in UIP or NSIP relative to CLD(−) according to the predefined threshold-based criteria (B, Methods). Clustering was performed on quantile-normalized NPX (log2) values using Euclidean distance and Ward's minimum variance linkage. Columns represent samples and rows represent proteins: the top annotation indicates UIP, NSIP, or CLD(−) group labels. When restricting the analysis to this elevated-protein set, samples clearly separated CLD(−) from ILD (UIP/NSIP) at the cohort level. RA, rheumatoid arthritis; ILD, interstitial lung disease; CLD(−), without chronic lung disease; UIP, usual interstitial pneumonia; NSIP, nonspecific interstitial pneumonia; NPX, normalized protein expression.
