# Supplementary material for: Blood protein biomarkers for rheumatoid arthritis patients complicated by interstitial lung disease
Source: Front Med (Lausanne). 2026 Aug 12;13:1890259. doi: 10.3389/fmed.2026.1890259 (PMC13509132; doi:10.3389/fmed.2026.1890259)
Supplement: Supplementary file 5 [file Data_Sheet_5.pdf]

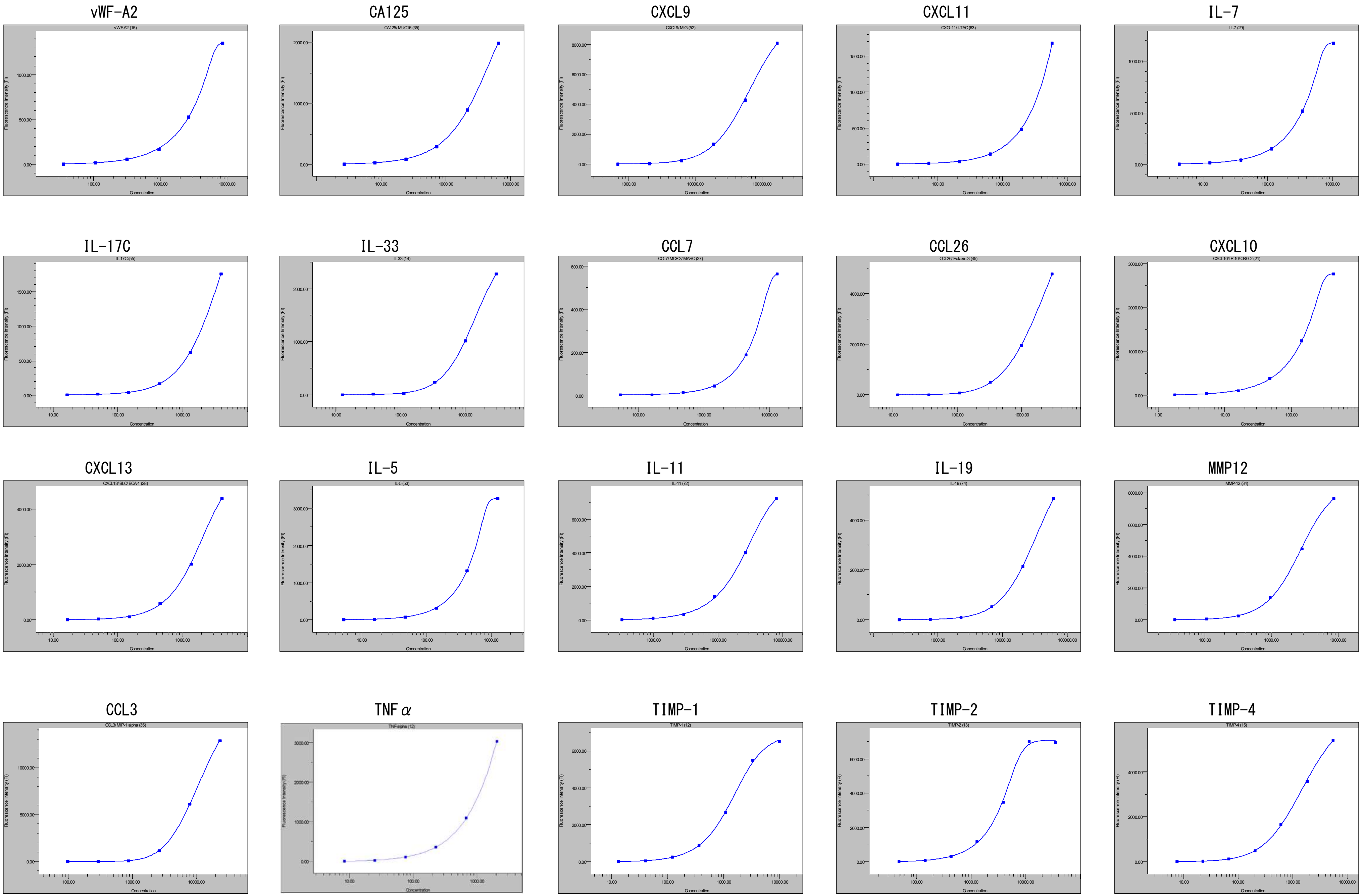


**Supplementary Figure S5. Standard curves for multiplex bead-based immunoassay of 20 candidate protein biomarkers.**
