# Supplementary material for: Blood protein biomarkers for rheumatoid arthritis patients complicated by interstitial lung disease
Source: Front Med (Lausanne). 2026 Aug 12;13:1890259. doi: 10.3389/fmed.2026.1890259 (PMC13509132; doi:10.3389/fmed.2026.1890259)
Supplement: Supplementary file 6 [file Data_Sheet_6.pdf]

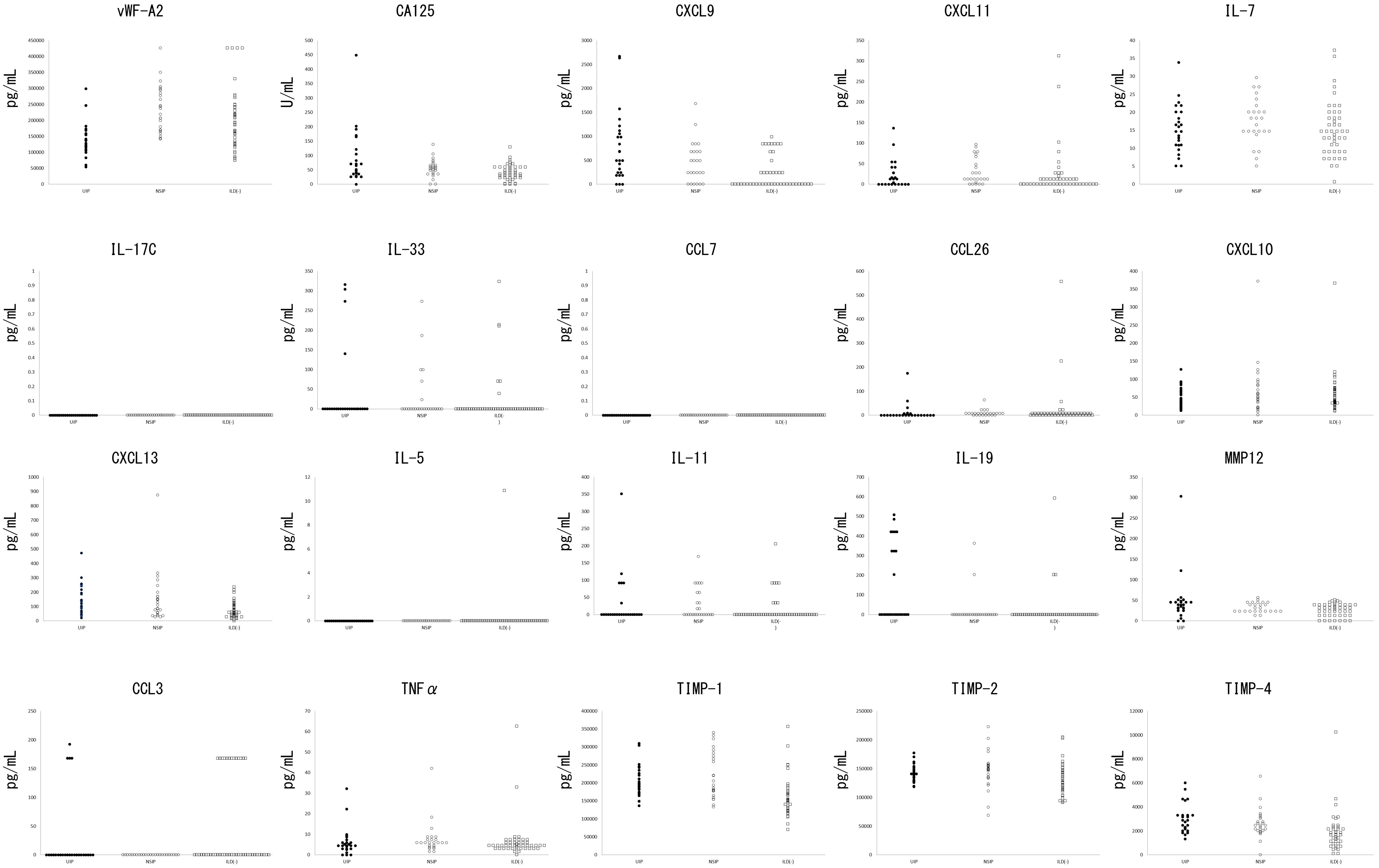

Supplementary Figure S6. Dot plots of protein biomarker concentrations in sera from RA subgroups. Dot plots showing protein concentrations in sera from RA patients with UIP, NSIP, and without CLD subgroups. RA, rheumatoid arthritis; CLD(−), without chronic lung disease; UIP, usual interstitial pneumonia; NSIP, nonspecific interstitial pneumonia.
