# Supplementary material for: Blood protein biomarkers for rheumatoid arthritis patients complicated by interstitial lung disease
Source: Front Med (Lausanne). 2026 Aug 12;13:1890259. doi: 10.3389/fmed.2026.1890259 (PMC13509132; doi:10.3389/fmed.2026.1890259)
Supplement: Supplementary file 7 [file Data_Sheet_7.pdf]

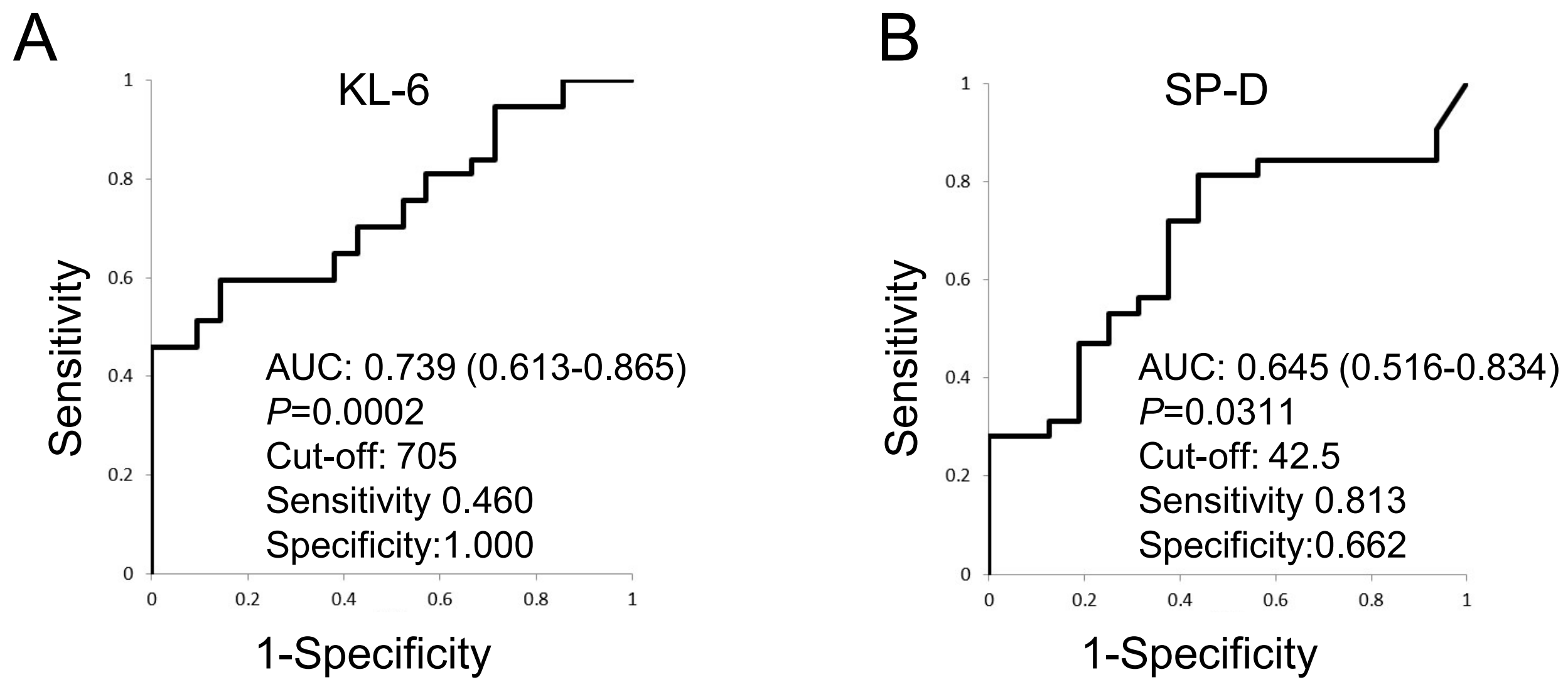

Supplementary Figure S7. ROC curves using KL-6 and SP-D for comparisons between RA ILD(+) and RA CLD(−). ROC curves for KL-6 (A) and CSP-D (B) are shown. The AUC values of the ROC curves with 95% confidence intervals and the optimized cut-off levels with specificities and sensitivities are described. RA: rheumatoid arthritis, ILD: interstitial lung disease, CLD: chronic lung disease, CLD(−): without CLD. ILD group includes usual interstitial pneumonia and nonspecific interstitial pneumonia groups. ROC: receiver operating characteristic, AUC: area under the curve.
